# Immune Thrombocytopenia Purpura Associated With COVID-19 Infection: A Challenging Diagnosis and Management

**DOI:** 10.7759/cureus.47433

**Published:** 2023-10-21

**Authors:** João Lemos, João Francisco Poças, Inês Castro, Liliana Ferreira Mota, Ana Correia de Oliveira

**Affiliations:** 1 Family Medicine, Unidade de Saúde Familiar (USF) Cedofeita, Porto, PRT

**Keywords:** patient-centred care, post-infection period, acute haemoptysis, interdisciplinary cooperation, immune thrombocytopenia purpura, covid-19

## Abstract

The COVID-19 pandemic has posed unprecedented challenges in the field of medicine. Among its diverse manifestations, immune thrombocytopenia purpura (ITP) has emerged as a complication associated with COVID-19 infection. This case report presents a 90-year-old Caucasian male with a history of COVID-19 infection who developed acute hemoptysis, thrombocytopenia, and purpura. The diagnosis of ITP confirmed through exclusion criteria and clinical evaluation, occurred several weeks after the initial COVID-19 diagnosis. Differential diagnoses, including drug-induced thrombocytopenia, were carefully considered and excluded.

The reported case highlights the importance of vigilance in identifying ITP as a potential complication of COVID-19 infection, even in the post-infection period. The timely initiation of appropriate treatment proved effective in managing the patient's condition. Collaboration among medical specialties facilitated comprehensive patient care, thereby reducing hospitalization periods.

This case report serves as a crucial alert to physicians, underlining the need for ongoing monitoring of COVID-19 complications, especially as testing dwindles. By fostering interdisciplinary cooperation, healthcare professionals can optimize patient outcomes and effectively manage the multifaceted challenges associated with COVID-19 infection.

## Introduction

Immune thrombocytopenic purpura (ITP), also known as idiopathic thrombocytopenic purpura, is an autoimmune disorder characterized by the presence of autoantibodies against platelet antigens, leading to thrombocytopenia [1]. The diagnosis of ITP is based on exclusion criteria and encompasses a range of clinical syndromes [2]. However, the identification of ITP can be challenging due to the diverse array of pathologies or drug side effects that can cause thrombocytopenia.

ITP can arise either as a primary autoimmune event or as a secondary condition associated with clinical disorders, including infections and cancer. Notably, there is a documented connection between ITP and certain oncologic diseases, such as chronic lymphocytic leukemia, as well as bacterial (e.g., *Helicobacter pylori*) and viral infections (e.g., hepatitis C virus, cytomegalovirus, varicella-zoster virus, human immunodeficiency virus) [2]. Additionally, several medications are also associated with thrombocytopenia, resulting in drug-induced thrombocytopenia (DiT). More recently, ITP has been linked to COVID-19 infection, both at the time of infection and in the weeks following disease onset. There have been reports of ITP diagnosis occurring as late as five weeks and even up to 125 days after the initial COVID-19 diagnosis [3-10]. This potential association prompted the British Society for Haematology to issue guidelines concerning treatment options for ITP cases in the context of COVID-19 infection [3]. Given that ITP is a rare and potentially life-threatening condition, it is crucial to be aware of this association to facilitate accurate diagnosis and provide optimal healthcare management.

## Case presentation

We reported a case of a 90-year-old Caucasian male with a medical history of hypertension, dyslipidemia, benign prostatic hyperplasia, and chronic anemia (hemoglobin baseline levels: 10.9g/dL to 11.5g/dL), in addition to being a carrier of a biological aortic valve prosthesis and a pacemaker. The patient's home medications included furosemide, atorvastatin, valsartan, warfarin, dutasteride, and tamsulosin. He regularly attended check-up appointments with his primary care physician without any reports of major complications or other medical problems. The patient received three doses of the Comirnaty® vaccine without experiencing any side effects, approximately three months prior to being diagnosed with COVID-19. At the time of diagnosis, the patient presented with a dry cough, mild dyspnea, and abnormal pulmonary auscultation (bilateral inferior crackles, and diffuse rustling vesicular breath sounds). He had received only supportive care and had been contacted by his family doctor. Following Portuguese guidelines available at that time, he was discharged from home isolation seven days after the onset of symptoms. However, he continued to experience dyspnea and cough. Twenty-six days after discharge, he was assessed and prescribed amoxicillin and clavulanic acid (875mg + 125mg), budesonide and formoterol (160µg/dose + 4.5µg/dose), and ipratropium bromide (20µg/dose). Approximately 72 hours after initiating the new medication, the patient experienced nausea with vomiting and severe hemoptysis, leading him to the emergency department.

**Figure 1:**
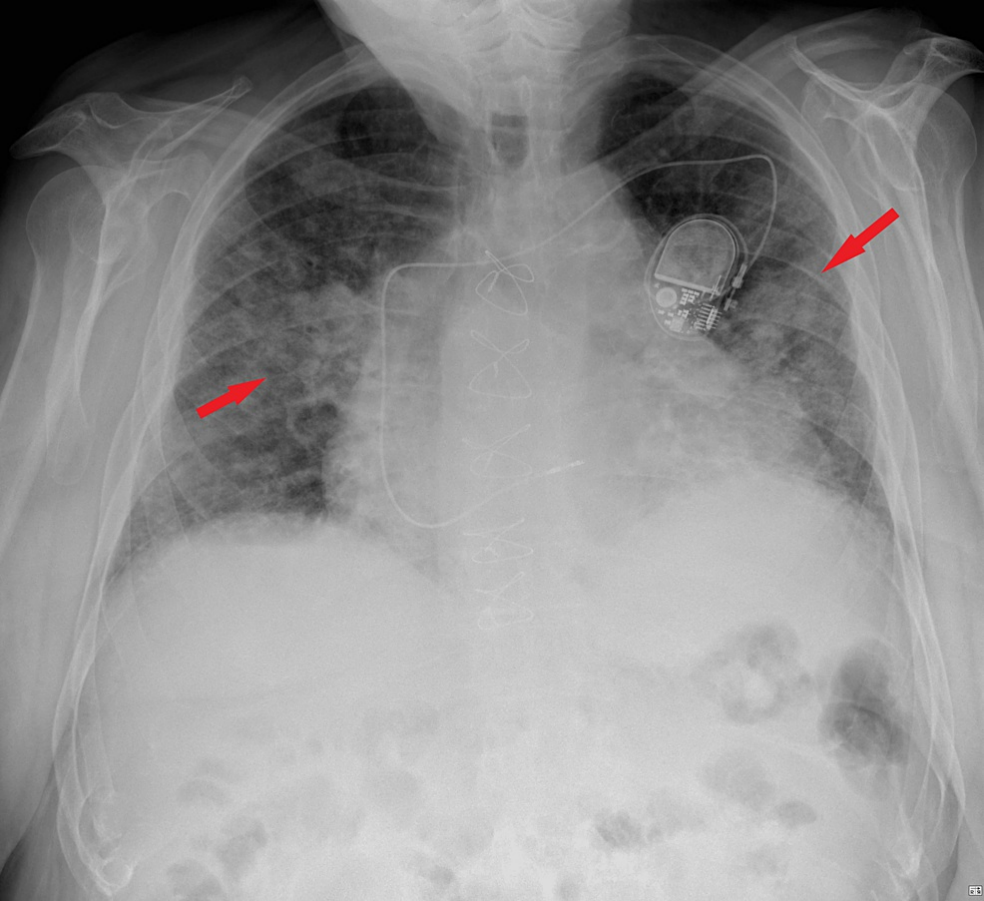
Chest x-ray during acute phase showing diffuse bilateral, infiltrative opacification patterns, sparing the pulmonary apex consistent with an acute diffuse alveolar hemorrhage (represented by the red arrows)

At the emergency department, the patient exhibited thrombocytopenia with a platelet count of less than 10x10^9^/L (reference range: 150-400x10^9^/L), as well as purpura lesions and a petechial rash on his lower limbs. He also presented with type one respiratory failure and stage one acute kidney injury. In the emergency department, the patient received fluid therapy, and supplementary oxygen via nasal cannula at a rate of 2L/minute, 200mg of hydrocortisone, followed by 40mg of prednisolone and 90g of intravenous immunoglobulin (IVIg). Additionally, he received two pools of platelet transfusions. The patient was admitted and received five days of prednisolone at a dose of 1mg/kg/day and two days of IVIg at a dose of 1g/kg/day. Although no clear signs of pulmonary infection were observed, the thoracic angiography and chest x-ray, indicated signs of alveolar hemorrhage (Figure 1). A blood smear revealed no abnormal platelets or schistocytes. Bone marrow flow cytometric immunophenotyping showed no relevant abnormalities. Direct and indirect antiglobulin tests, as well as tests for Kell antigen and Cw antigen, were negative. Serological tests for hepatitis C and B virus, human immunodeficiency virus one and two, and venereal disease research laboratory test (VDRL) were negative. The sputum culture yielded no growth of common bacteria, and the patient had no history of *H. pylori* infection. Thyroid function tests were within the normal range.

The patient denied any family history of bleeding disorders or prior thrombocytopenia and reported no use of tobacco, alcohol, or recreational drugs. During hospitalization, anticoagulant therapy was discontinued, but the patient continued his regular medication regimen and completed the remaining seven days of treatment with amoxicillin and clavulanic acid. Within five days, his platelet count increased from less than 10x10^9^/L to 175x10^9^/L (Table 1). He was discharged five days after admission with a diagnosis of ITP and scheduled for follow-up with his primary care physician. Table 1 presents the progression of blood analysis during the patient's treatment.

**Table 1:** Progression of blood analysis during the patient's treatment. In the table is highlighted the progression in absolute platelet count. This improvement becomes evident within a mere 24-hour window after the initiation of the therapeutic intervention.

Test (units of measurement)	Emergency department	Day One	Day Two	Day three	Day Four	Day Five (Discharge)	Reference range
Haemoglobin (g/dL)	10.8	9.0	8.1	7.8	8.0	8.5	13.5-17.5
Haematocrit (%)	32.2	27.1	24.1	23.5	23.2	25.8	41.0-53.0
Mean Corpuscular Volume (µm^3^)	93.8	93.9	96.0	94.8	93.2	94.9	80.0-100
Mean corpuscular haemoglobin (pg)	31.1	315	32.3	31.5	32.1	31.3	25.0-35.0
Mean corpuscular haemoglobin concentration (g/dL)	33.5	33.2	33.6	33.2	34.5	32.9	31.0-36.0
RDW (%)	13.8	13.5	13.6	13.6	13.6	13.7	11.5-14.5
Leucocytes (× 10^9^ /L)	10.45	6.38	7.22	5.90	5.06	5.00	4.5-11.0
Platelets (× 10^9^ /L)	<10	35	87	119	144	175	150-400
Creatinine (mg/dL)	1.36	1.36	1.69	1.64	1.58	-	0.6-1.2
Total Bilirubin (mg/dL)	0.70	-	0.75	0.75	0.90	-	0.1-1.0
Lactate dehydrogenase (U/L)	-	331	373	-	332	-	45.0-90.0
Ferritin (ng/mL	-	450	-	-	430	-	12.8-454
Sodium (mEq/L)	140	139	138	-	140	-	135.0-146.0
Potassium(mEq/L)	3.72	3.87	4.20	-	4.09	-	3.5-5.0
Chlorates (mEq/L)	103	104	103	-	-	-	95.0-105.0
International normalized ratio	2.56	1.50	-	-	-	-	0.8-1.0
Prothrombin time (s)	39.0	16.9	-	-	-	-	<12
Activated partial thromboplastin time (s)	29.2	30.9	-	-	-	-	<28

## Discussion

This report presents a case of ITP diagnosed following an episode of acute hemoptysis. While not a common presentation, this can be life-threatening and poses a challenge for emergency room management. The most likely trigger for the development of ITP in this case was the infection of COVID-19. As demonstrated in this report, ITP can manifest several weeks after the initial diagnosis of COVID-19. While initial reports primarily linked ITP to vaccination, recent literature has also reported associations between ITP and COVID-19 infection [3-10]. In the presented case, the time gap between the initial positive COVID-19 test and the diagnosis of ITP was approximately five weeks, which is compatible with another report of COVID-19-associated ITP [7-10]. Viral infections, including COVID-19, have been implicated in various autoimmune mechanisms associated with ITP. The inflammatory state induced by COVID-19 and the underlying immune dysregulation appears to have an impact on the production of platelet antibodies [3,8].

In this case, other conditions related to thrombocytopenia needed to be excluded. Hemolytic diseases were ruled out by the blood work and normal blood smear, which showed no new relevant changes in total bilirubin, ferritin, or other laboratory results and showed no schistocytes, or other abnormalities indicative of hemolysis. Although hemophagocytic lymphohistiocytosis (HLH) has been associated with COVID-19 infection, the patient did not present with fever, splenomegaly, hepatomegaly, or high levels of ferritin at the time of diagnosis. Furthermore, the patient's response to treatment makes HLH extremely unlikely.

DiT was also considered due to the introduction of amoxicillin/clavulanic acid. While rare, cases of amoxicillin-related thrombocytopenia have been reported in the literature [11]. According to international guidelines, the interval between initiation of a new drug and DiT is 5-14 days [3,12]. In this case, the patient experienced symptoms just three days after the first administration of the drug. Although earlier onset is possible with previous exposure, there is no record in the patient's medical history of prior administration of amoxicillin and clavulanic acid. Additionally, guidelines establish additional clinical criteria for diagnosing DiT such as the resolution of thrombocytopenia upon discontinuing the drug, the absence of any other identifiable causes, and lack of reoccurrence upon reexposure of the candidate drug [12]. In this case, none of these criteria were met. The patient continued to use all his medications and completed the full seven-day course of amoxicillin/clavulanic acid, rendering DiT less likely [12,13].

The favorable response to treatment, along with negative viral serologies, further supports the diagnosis of ITP induced by COVID-19 infection. Treatment adhered to guidelines provided by scientific associations and consisted of corticosteroids, specifically prednisolone at a dose of 1mg/kg/day. Additionally, due to the patient's condition during the initial days, IVIg at a dose of 1g/kg/day was administered for two days and subsequently discontinued upon observing clinical and analytical improvement [3]. Given the patient's instability in the emergency department, two pools of platelets were transfused. The applied treatment proved effective, leading to the patient's discharge five days after admission. Subsequent follow-up was arranged with the patient's family doctor at the health center. This collaboration among different medical specialties becomes crucial in managing these complex cases. The interdisciplinary approach ensures that patients benefit from a comprehensive and integrated healthcare strategy, addressing the multifaceted aspects of COVID-19 and its associated complications [14].

## Conclusions

In conclusion, physicians should maintain a high level of awareness regarding the potential for delayed complications of COVID-19 infection. As demonstrated in this report, ITP is a potentially life-threatening complication that can manifest several weeks after the initial diagnosis of COVID-19. By fostering collaboration between medical specialties, healthcare providers can effectively manage these complications, enhancing patient outcomes and minimizing the impact of the disease.

## References

[1] Neunert C, Terrell DR, Arnold DM (2019). American Society of Hematology 2019 guidelines for immune thrombocytopenia. Blood Adv.

[2] Cines DB, Bussel JB, Liebman HA, Luning Prak ET (2009). The ITP syndrome: pathogenic and clinical diversity. Blood.

[3] Pavord S, Thachil J, Hunt BJ (2020). Practical guidance for the management of adults with immune thrombocytopenia during the COVID-19 pandemic. Br J Haematol.

[4] Pedroso A, Frade L, Trevas S, Correia MJ, Esteves AL (2020). Immune thrombocytopenic purpura - different presentations in two COVID-19 patients. Cureus.

[5] Watts A, Raj K, Gogia P, Toquica Gahona CC, Porcelli M (2021). Secondary immune thrombocytopenic purpura triggered by COVID-19. Cureus.

[6] Santhosh S, Malik B, Kalantary A, Kunadi A (2022). Immune thrombocytopenic purpura (ITP) following natural COVID-19 infection. Cureus.

[7] Lee EJ, Liu X, Hou M, Bussel JB (2021). Immune thrombocytopenia during the COVID-19 pandemic. Br J Haematol.

[8] Kewan T, Gunaratne TN, Mushtaq K, Alayan D, Daw H, Haddad A (2021). Outcomes and management of immune thrombocytopenia secondary to COVID-19: Cleveland clinic experience. Transfusion.

[9] Alharbi MG, Alanazi N, Yousef A, Alanazi N, Alotaibi B, Aljurf M, El Fakih R (2022). COVID-19 associated with immune thrombocytopenia: a systematic review and meta-analysis. Expert Rev Hematol.

[10] Davoodian A, Umeh C, Novatcheva E, Sassi GP, Ahaneku H, Kundu A (2021). Severe immune thrombocytopenia post-COVID- 19: a case report. Cureus.

[11] Mansour H, Saad A, Azar M, Khoueiry P (2014). Amoxicillin/clavulanic acid-induced thrombocytopenia. Hosp Pharm.

[12] Bakchoul T, Marini I (2018). Drug-associated thrombocytopenia. Hematology Am Soc Hematol Educ Program.

[13] Marini I, Uzun G, Jamal K, Bakchoul T (2022). Treatment of drug-induced immune thrombocytopenias. Haematologica.

[14] Ciemins EL, Brant J, Kersten D, Mullette E, Dickerson D (2016). Why the interdisciplinary team approach works: insights from complexity science. J Palliat Med.

